# Radioprotective effects and mechanism of HL-003 on radiation-induced salivary gland damage in mice

**DOI:** 10.1038/s41598-022-12581-y

**Published:** 2022-05-19

**Authors:** Jingming Ren, Rong Huang, Yanjie Li, Ruiyang Chen, Hongqi Tian, Chenlu Liu

**Affiliations:** 1 Tianjin Key Laboratory of Radiation Medicine and Molecular Nuclear Medicine, Institute of Radiation Medicine, Peking Union Medical College and Chinese Academy of Medical Science, Tianjin, 300192 China; 2 Department of Oral Medicine, Tianjin Stomatological Hospital, School of Medicine, Nankai University, Tianjin, 300041 China; 3 Tianjin Key Laboratory of Oral and Maxillofacial Function Reconstruction, Tianjin, 300041 China; 4 KeChow Pharma, Inc., Shanghai, 201203 China

**Keywords:** Cancer, Cell biology

## Abstract

Ionizing radiation (IR) can cause damage to the structure and function of salivary glands. Our research group independently synthesized the ROS scavenger, HL-003. The aim of this study was to explore the protective effects and underlying mechanisms of HL-003 on radiation-induced salivary gland injury. Salivary flow rate measurement, H&E staining, immunohistochemistry, FRAP, TUNEL, and western blotting were used to evaluate the radioprotective effect on salivary glands. The results showed that HL-003 protected the salivary secretion function by protecting the AQP-5 protein, on the salivary epithelial cell membrane, from IR damage. HL-003 reduced oxidative stress in the salivary gland by regulating the expression of ROS-related proteins NOX4, SOD2, and 8-OHdG. Furthermore, HL-003 downregulated the expression of p-p53, Bax, caspase 3, and caspase 9, and upregulated the expression of Bcl-2, suggesting that it could inhibit the activation of p53 to reduce cell apoptosis. In conclusion, HL-003 is an effective radioprotector that prevents damage of the radiation-induced salivary gland.

## Introduction

Head and neck cancer (HNC) is the seventh most common occurring malignancy in the world^1^. The global incidence of HNC was more than nine million in 2020 (accounting for 4.9% of all cancer sites)^2^, and the annual incidence in China was approximately 142,000 cases (accounting for 3.1% of all cancer sites)^3^. Radiotherapy is the main treatment used in patients with HNC and can significantly prolong the survival time of patients; however, it inevitably damages the normal tissues near the tumor, resulting in patients with dysphagia and impaired taste. Xerostomia is one of the most frequent complications that results from salivary gland dysfunction^4^; it is a common and irreversible health problem in patients with HNC^5^. It is characterized by a slowly progressive course, which causes difficulty to patients in swallowing, speaking, and chewing, affecting the quality of life and even interrupting the course of treatment^6,7^.

Radiation-induced salivary gland dysfunction is mainly manifested as decreased salivary secretion, acinar and ductal cell damage, oxidative stress, and apoptosis^8^. Ionizing radiation (IR) produces reactive oxygen species (ROS) in biological tissues^9^. Excessive ROS can considerably damage the acinar and ductal cells of the salivary glands, and destroy cellular components such as proteins, DNA, and lipids^10^. Under physiological conditions, intracellular antioxidants, such as glutathione peroxidase, can clear ROS to reduce oxidative stress, a particular state of cellular stress. However, the level of ROS in the salivary glands increases significantly after IR. Studies have shown that ROS are involved in regulating a variety of cellular physiological functions, including energy metabolism, stress reactions, growth, and proliferation. Superfluous ROS can affect intracellular signal transduction pathways, causing cell apoptosis and organ dysfunction^9^. Due to the slow turnover of salivary gland cells, it is believed that the salivary glands are not sensitive to radiation^11^. However, many studies have shown that salivary gland dysfunction is an acute response that occurs within a few days after IR^12–15^, thus, finding an effective radioprotector is crucial.

Presently, many natural products and synthetic compounds are considered to have radioprotective effects. Recent studies have shown that natural products such as epigallocatechin gallate^16^, curcumin^17^, and resveratrol^13^ can significantly scavenge free radicals and improve the body's immune function in the body. However, the extraction and purification of natural products are very difficult and challenging to apply in clinical practice. Amifostine, a radioprotective drug, is currently the only antioxidant approved by the United States Food and Drug Administration. It acts as a protective agent that protects normal cells, and can reduce xerostomia in HNC patients undergoing radiation therapy by scavenging free radicals, preferentially locating and accumulating in the salivary glands^18^. Studies have shown that amifostine has a good protective effect on the salivary glands, but does not protect tumor cells^19^. Unfortunately, the disadvantage of amifostine is that it has major adverse effects, including hypocalcemia, hypotension, and severe digestive tract reaction^20^. The route of administration and high cost limit the clinical application of amifostine.

HL-003 is an ROS scavenger that was independently developed by our research group. The research results showed that HL-003 not only has a similar anti-oxidative stress performance to amifostine, but also overcomes the adverse reactions of amifostine and has better protection^21^. HL-003 could protect against radiation-induced intestinal injury and inhibit cisplatin-induced nephrotoxicity^21,22^. Therefore, the purpose of this study was to identify an oral, safe, and efficient radioprotector and explore the radioprotective effect of HL-003 on the salivary glands of HNC patients. We used a mouse local irradiation model to evaluate the role of HL-003 in alleviating the salivary gland damage caused by IR and explored its possible influence on in vivo mechanisms.

## Materials and methods

### Animals

Twenty-four 6-week-old male, C57BL/6 mice weighing 20 ± 2 g were purchased from Huafukang Bioscience Co. (Beijing, China). The mice were maintained under pathogen-free conditions at the experimental animal center of the Institute of Radiation Medicine (IRM), Chinese Academy of Medical Sciences (CAMS). The animal center controls the light/dark cycle, and provides clean water and a standard diet.

### Irradiation and drug treatment

We used an intraperitoneal injection of 50 mg/kg pentobarbital sodium to anesthetize all animals. The salivary glands of mice were irradiated with a single dose of 15 Gy with an RS2000 Pro X-ray irradiator (Rad Source Technologies, Inc., Suwanee, GA, USA) at a dose rate of 2.0 Gy/min. Mice in the control group were sham-irradiated.

Mice were divided into 4 groups (6 mice per group): the control group, IR group (15 Gy), IR + HL-003 (15 Gy + 1000 mg/kg) group, and IR + amifostine (15 Gy + 200 mg/kg) group. HL-003 was dissolved in saline and intragastrically administered 4 h before IR. Amifostine (BioPharmaMatrix, Jiangsu, China) was administered intraperitoneally given 30 min before IR. The mice were sacrificed 2 weeks after the IR. Salivary gland tissues were obtained for biochemical and pathological analyses.

### Salivary flow rate measurement

At 3, 7, and 10 days after IR, each mouse was intraperitoneally injected with pilocarpine hydrochloride (0.5 mg/kg, Sigma) to stimulate saliva. The mice were placed in a vertical position (head-down), and saliva was collected in the oral cavity for 10 min with cotton balls that were weighed in advance. To prevent moisture loss, the cotton balls were weighted immediately after the experiment using an electronic balance. The salivary specific gravity was assumed to be 1.0 g/cm^3^. The salivary flow rate (SFR) was calculated as the total volume of saliva divided by the collection time (μL/min).

### Histopathology

Mice were euthanized by cervical dislocation. Both the salivary glands of mice were excised, and the right salivary glands were collected in freezing tubes and stored in liquid nitrogen for subsequent experiments. The left salivary glands were soaked in freshly prepared 4% formalin solution, embedded in paraffin and sectioned. Sections were 3–5 μm in thickness and dewaxed with xylene. The sections were stained with hematoxylin and eosin (H&E) and analyzed under a microscope (Olympus, DP26). Three areas were randomly selected from each section. The results of H&E staining were independently evaluated by two pathologists.

### Immunohistochemistry

The sections were deparaffinized with xylene and rehydrated in ethanol. Used EDTA antigen retrieval solution was used for antigen retrieval, and endogenous peroxidase was blocked with serum. The sections were incubated with aquaporin 5 (AQP-5; 1:1000 dilution; ab78486; Abcam, Cambridge, MA, USA), superoxide dismutase 2 (SOD2; 1:1000 dilution; 24127-1-AP; Proteintech, Wuhan, China), 8-hydroxy-2’-deoxyguanosine (8-OHdG; 1:50 dilution; ab48508; Abcam), Caspase 9 (1:300 dilution; ab202068; Abcam), and gamma H2AX (1:5000 dilution; ab81299; Abcam), followed by incubation with the secondary antibodies. Furthermore, a DAB kit was used to stain the cells, which showed brown-yellow are positive cells. Three areas were randomly selected from each section. Blind researchers used ImageJ software to quantify the proportion of observed positive areas in immunohistochemistry. A microscope (Olympus, DP26) was used to observe the cells at × 200 magnification.

### Ferric reducing ability of plasma assay

The total antioxidant capacity (T-AOC) assay kit (S0116; Beyotime Biotechnology, Nanjing, China) with ferric reducing ability of plasma (FRAP) was used to detect T-AOC in salivary glands. Tissue samples were dissolved in cold PBS solution and ultrasonicated to adequately break the tissues and release its antioxidants. The supernatant of the samples was collected after centrifugation. According to the assay kit, 2,4,6-tri(2-pyridyl)-s-triazine (TPTZ) diluent, TPTZ solution and detection buffer were mixed together in a ratio of 10: 1: 1 as the working FRAP reagent. The working FRAP reagent (180 μL) was added to a 5 μL sample and incubated at 37 °C for 5 min. The absorbance was measured at 593 nm. The total antioxidant capacity of the samples was calculated using the FeSO_4_ standard curve.

### Malondialdehyde (MDA) assay

We used the lipid peroxidation MDA assay kit (S0131S; Beyotime Biotechnology) to detect MDA in salivary glands. Tissue samples were dissolved in lysis buffer (P0013; Beyotime Biotechnology) and ultrasonicated to adequately break the tissues. The supernatant of the samples was collected after centrifugation. Prepared the working MDA reagent according to the manufacturer’s instructions. The working MDA reagent (200 μL) was added to a 100 μL sample and heated for 15 min. The absorbance was measured at 532 nm. The MDA concentration of the samples was calculated according to the MDA standard curve.

### TUNEL assay

A TdT-mediated dUTP Nick End Labeling (TUNEL) Apoptosis Detection Kit (C1086; Beyotime Biotechnology) was used to detect apoptosis in salivary glands. After paraffin sections were dewaxed with xylene, added proteinase K working solution was added to cover the tissue and incubated at 37 °C for 20 min. The sections were washed three times with PBS (pH 7.4). According to the assay kit, TdT and dUTP were mixed together in a ratio of 1: 9 as the TUNEL reaction solution. The tissue sections were covered with TUNEL reaction solution, placed in a humid box, and incubated at 37 °C for 2 h. After washing thrice with PBS, the sections were stained with DAPI solution (C02-04002, Bioss Antibodies, Beijing, China) in the dark for 10 min at room temperature. Finally, a fluorescence quenching agent (S2110, Solarbio, Beijing, China) was used to seal the sections and evaluate the number of TUNEL-positive apoptotic cells using a fluorescence microscope (Olympus, BX51) at × 200 magnification. The number of green fluorescent cells in these areas were considered to be the number of TUNEL-positive cells.

### Western blotting

Salivary gland tissues were homogenized in cold radioimmunoprecipitation assay lysis (RIPA) buffer, which was mixed with a protease inhibitor cocktail and phenylmethylsulfonyl fluoride (PMSF). Samples were incubated at room temperature for 30 min, and centrifuged at 12,000 rpm for 15 min at 4 °C. Then The supernatant was collected and boiled at 100 °C for 10 min.

We used 10% SDS PAGE gels to separate equal amounts of proteins, and transferred the proteins using polyvinylidene difluoride membranes. Membranes were blocked with 10% skim milk for 2 h at room temperature. The membranes were then incubated with primary antibodies against NADPH oxidase 4 (NOX4; 1:2000 dilution; 14347-1-AP; Proteintech), Bax (1:5000 dilution; ab3191; Abcam), Bcl-2 (1:1000 dilution; ab182858; Abcam), caspase 3 (1:2000 dilution; ab184787; Abcam), Phospho-p53 (1:2000 dilution; P04637; CST, MA, USA), and GAPDH (1:10,000 dilution; HRP-60004; Proteintech). All blots were detected using ECL chemiluminescence reagents and analyzed using the Image Lab software.

### Ethical approval

The study was carried out in compliance with the ARRIVE guidelines. We confirm that all experiments were performed in the National Institutes of Health guide for the care and use of Laboratory animals. And the experiments were approved by the Institutional Animal Ethics Committee of the IRM, CAMS (No. 2017053).

### Statistical analysis

Data analysis was conducted using the GraphPad Prism version 8 software. Differences between groups were analyzed using the t-tests and nonparametric tests. All data were expressed as mean ± standard error of the mean. Statistically significant was sat at p < 0.05.

## Results

### HL-003 prevents salivary dysfunction

**Figure 1:**
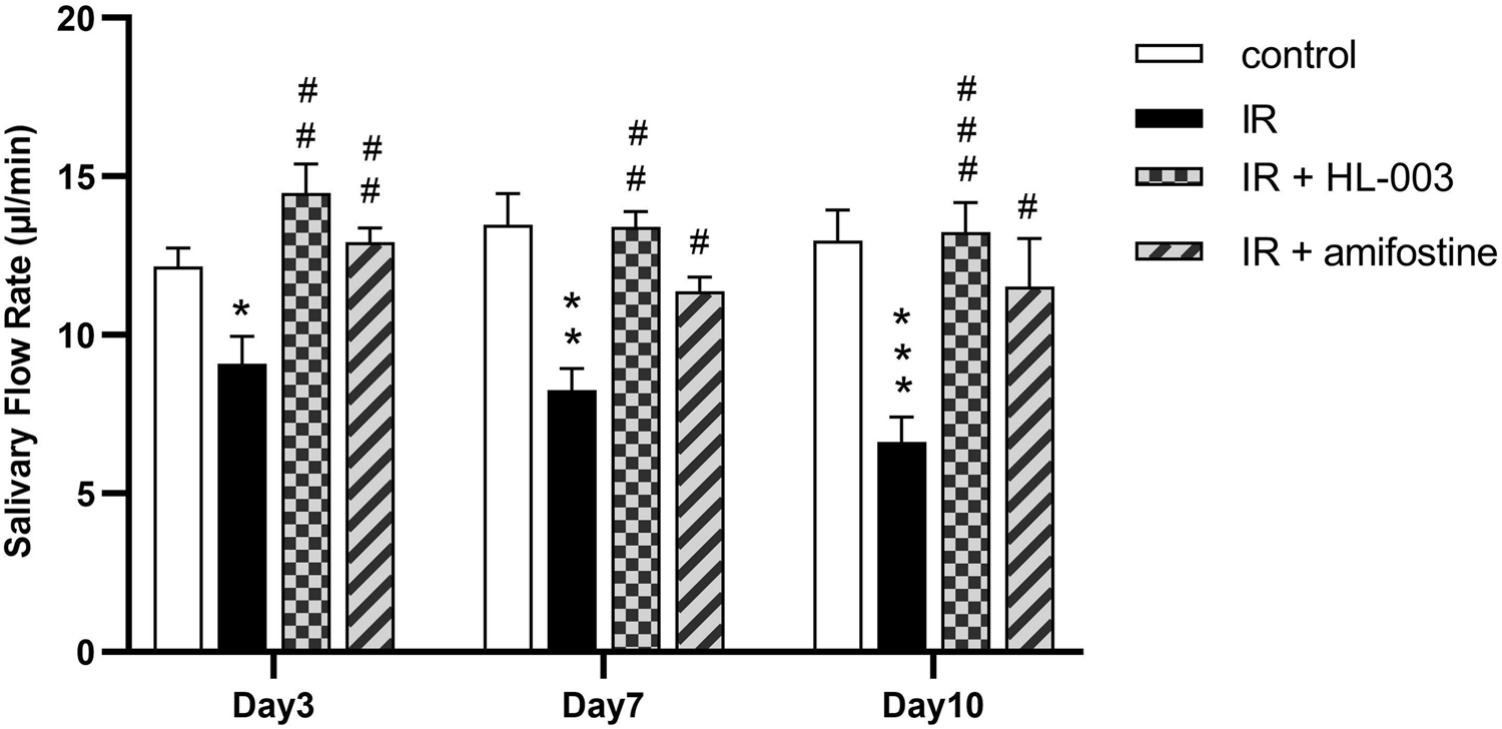
HL-003 prevents salivary function. The salivary flow rate was measured. Each bar shows the mean ± SEM; *p < 0.05, **p < 0.01, ***p < 0.001, compared with the control group; ^#^p < 0.05, ^##^p < 0.01, ^###^p < 0.001, compared with the IR group (n = 6 per group).

To determine whether HL-003 affects salivary function, the SFR was determined. The SFR in the IR group significantly decreased in a time-dependent manner compared with that in the control group. HL-003 and amifostine effectively prevented this decrease in the SFR (Fig. 1). However, there was no significant difference between the IR + HL-003 and IR + amifostine groups at 3, 7, and 10 days after IR. In particular, there were no significant differences in water intake, food intake, or body weight among the mice (Tables S1–S3).

### HL-003 alleviates salivary gland damages

**Figure 2:**
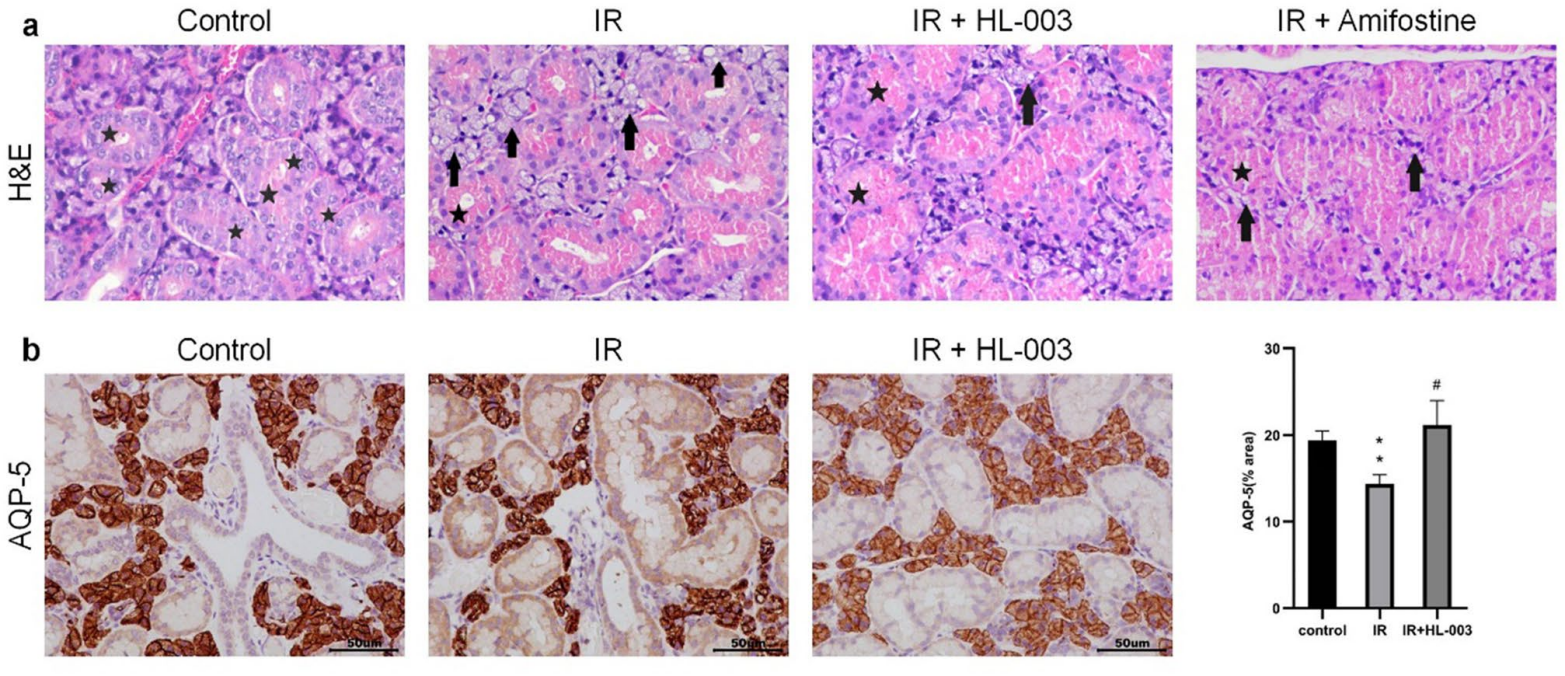
HL-003 alleviates damage of salivary glands. (**a**) Representative H&E staining of salivary gland structure. ★, zymogen granule; →, vacuolization. (**b**) Representative immunohistochemical images show salivary epithelial cells (AQP-5 staining) in the salivary glands. Scale bars represent 50 μm. Each bar shows the mean ± SEM; **p < 0.01, compared with the control group; ^#^p < 0.05, compared with the IR group (n = 6 per group).

Histological changes in salivary glands are shown by H&E staining. The control group had normal acinar cells with well-defined lobules. The ductal cells were well-developed, and abundant zymogen granules were observed in the duct. At 2 weeks, acinar cells with cytoplasmic vacuoles were observed in the IR group, which manifested as cytoplasmic vacuolization. However, there were no clear pathological changes in the interstitium. In contrast, histological analysis revealed that HL-003 and amifostine-treated groups had fewer vacuoles, clearer lobular structures, and more zymogen granules than the IR group (Fig. 2a).

We further examined the expression of the salivary epithelial marker, AQP-5, by immunohistochemistry. Compared to the control group, results revealed that the expressions of AQP-5 were lower in the IR group (Fig. 2b). In the HL-003 treated group, the staining intensity of AQP-5 was similar to that of the control group. These results suggest that HL-003 can protect the salivary epithelium from irradiation damage.

### HL-003 reduces oxidative stress

**Figure 3:**
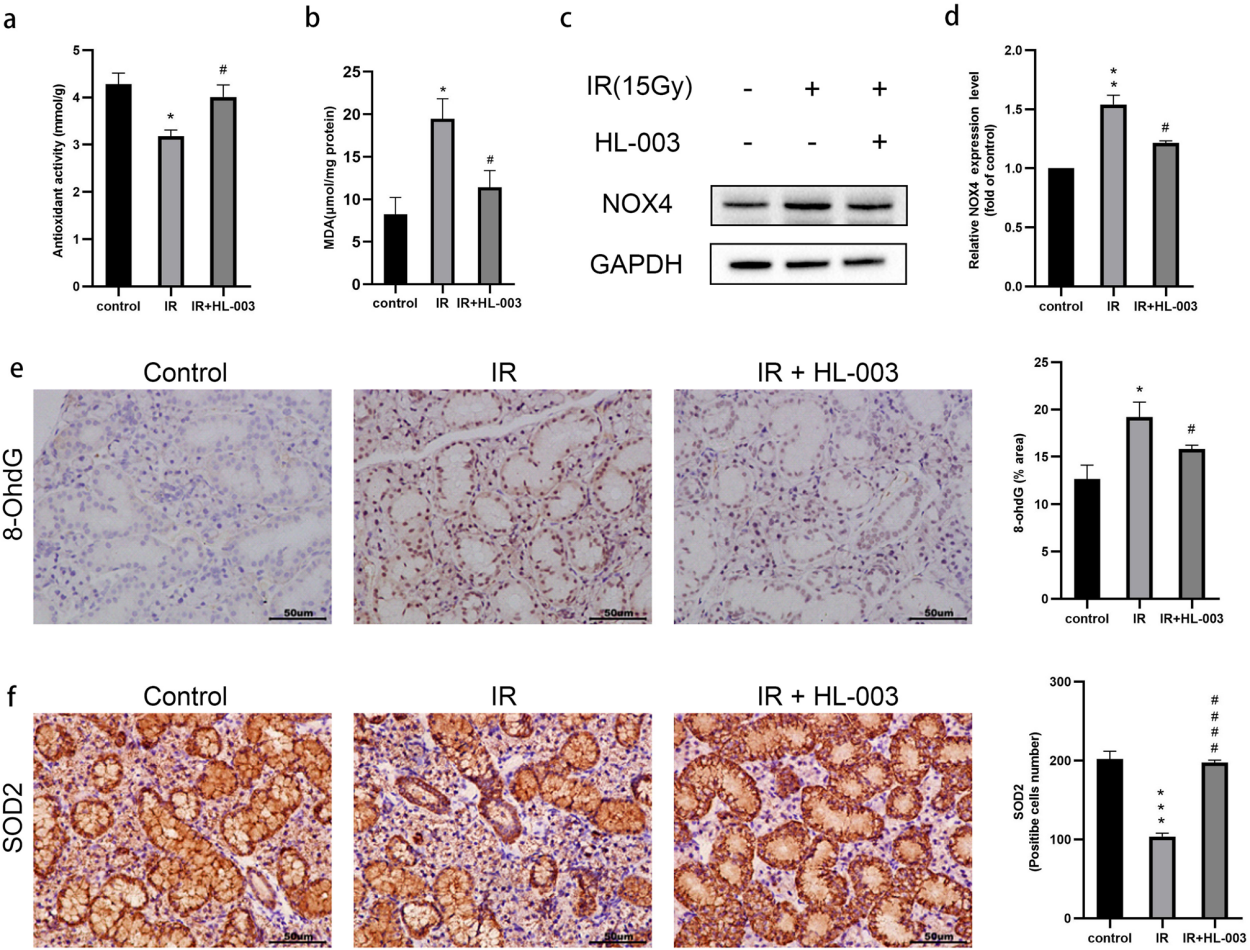
HL-003 reduces oxidative stress. (**a**) Total antioxidant capacity levels were determined. (**b**) The concentration of MDA was determined. (**c,d**) Western blot analysis of NOX4. Relative quantification of the expression of NOX4 is shown. (**e,f**) Representative immunohistochemical images of 8-OHdG and SOD2. Scale bars represent 50 μm. Relative quantitative analysis is shown. Each bar shows the mean ± SEM; *p < 0.05, **p < 0.01, ***p < 0.001, compared with the control group; ^#^p < 0.05, ^####^p < 0.0001, compared with the IR group (n = 6 per group).

It is common that radiation can enhance ROS production and cause salivary gland damage. Excessive ROS levels may cause DNA damage^9,10^. We examined T-AOC levels using FRAP. The experimental results showed that the T-AOC levels were decreased in the IR group compared with the control group (Fig. 3a). In addition, we detected the concentration of MDA, a lipid peroxidation product. Ionizing radiation induced a significant increase of MDA in salivary glands, while the concentration of MDA in the IR + HL-003 group could reduce (Fig. 3b).

To explore the relationship between oxidative stress and the radiation protection effect of HL-003, NOX4 was examined by western blotting, and the levels of 8-OHdG and SOD2 were measured by immunohistochemistry. IR induced an increase in NOX4 protein expression (Fig. 3c,d). Immunohistochemistry results revealed that 8-OHdG was strongly positive in the IR group compared to that in the control group. However, the levels of 8-OHdG were significantly lower in the IR + HL-003 group than in the IR group. The number of SOD2 positive cells in the IR + HL-003 group was higher than that in the IR group (Fig. 3e,f).

### HL-003 protects salivary glands from IR-induced DNA damage

**Figure 4:**
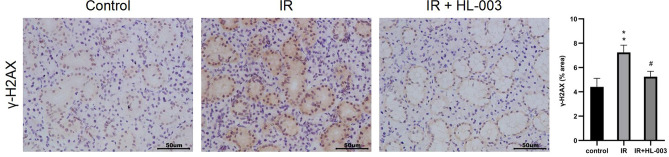
HL-003 protects salivary glands from IR-induced DNA damage. Immunohistochemical images of γ-H2AX are shown. Scale bars represent 50 μm. Each bar shows the mean ± SEM; **p < 0.01, compared with the control group; ^#^p < 0.05, compared with the IR group (n = 6 per group).

We used immunohistochemistry to analyze the level of γ-H2AX, a marker for DNA double-strand breaks (DSBs)^23^, and investigated whether HL-003 can protect DNA from irradiation damage. Compared with the control group, the expression of γ-H2AX was increased in the salivary glands of the IR group, whereas treatment with HL-003 markedly improved γ-H2AX expression (Fig. 4). These results suggest that HL-003 can reduce oxidative stress and DSBs in the salivary glands of irradiated mice.

### HL-003 suppresses the apoptotic signaling pathway

**Figure 5:**
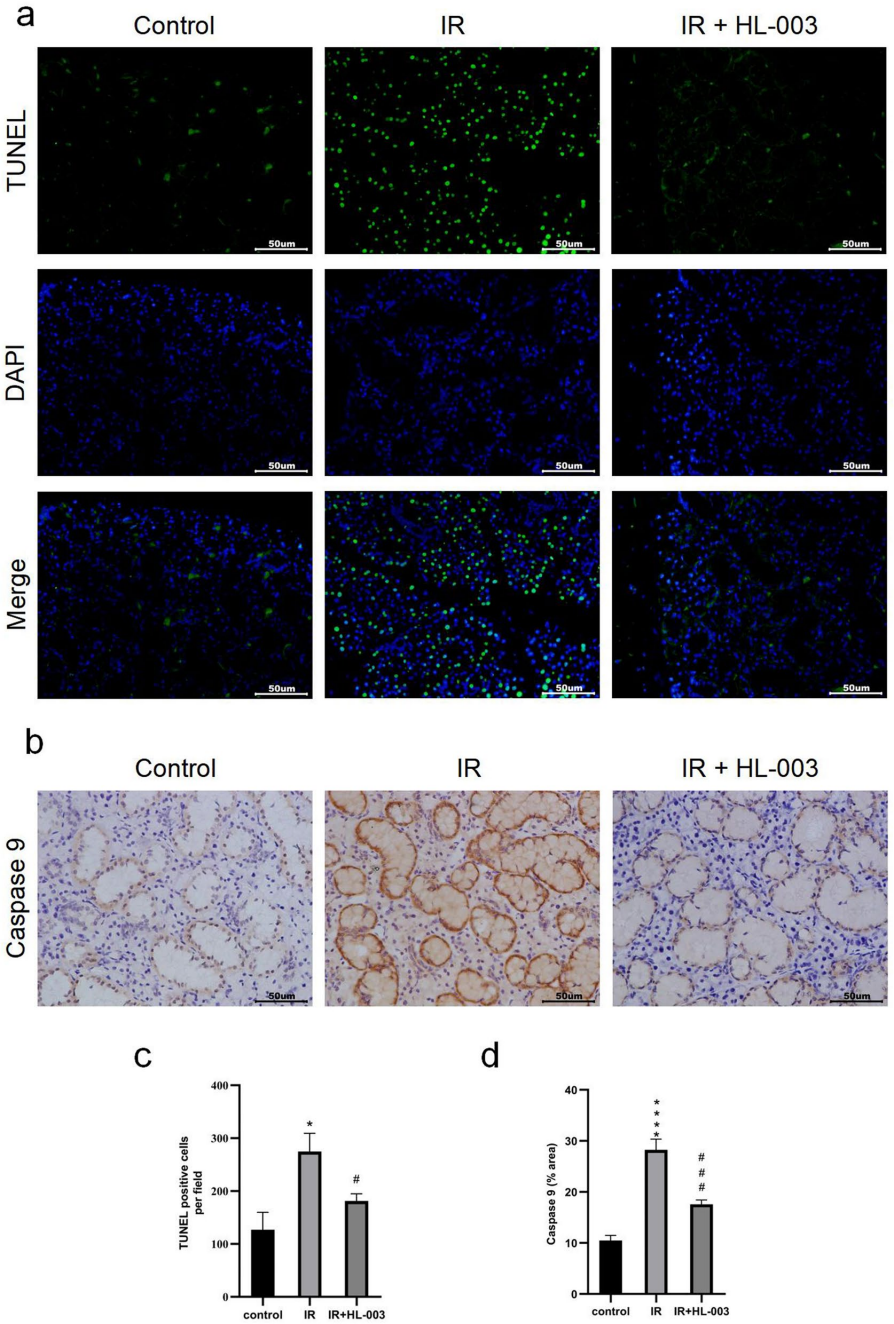
HL-003 suppresses the apoptotic signaling pathway. (**a**) Apoptosis was analyzed using TUNEL assay. (**b**) Representative immunohistochemical images of caspase 9. (**c**,**d**) Relative quantitative analysis is shown. Scale bars represent 50 μm. Each bar shows the mean ± SEM; *p < 0.05, ****p < 0.0001, compared with the control group; ^#^p < 0.05, ^###^p < 0.001, compared with the IR group (n = 6 per group).

TUNEL staining of salivary gland tissues was performed to detect apoptosis. Positive cells included acinar, ductal, and endothelial cells. The results showed that the number of apoptotic cells in the IR group was markedly higher than that in the control group (Fig. 5a,c). In contrast, the number of apoptotic cells was lower in the IR + HL-003 group than in the IR group.

**Figure 6:**
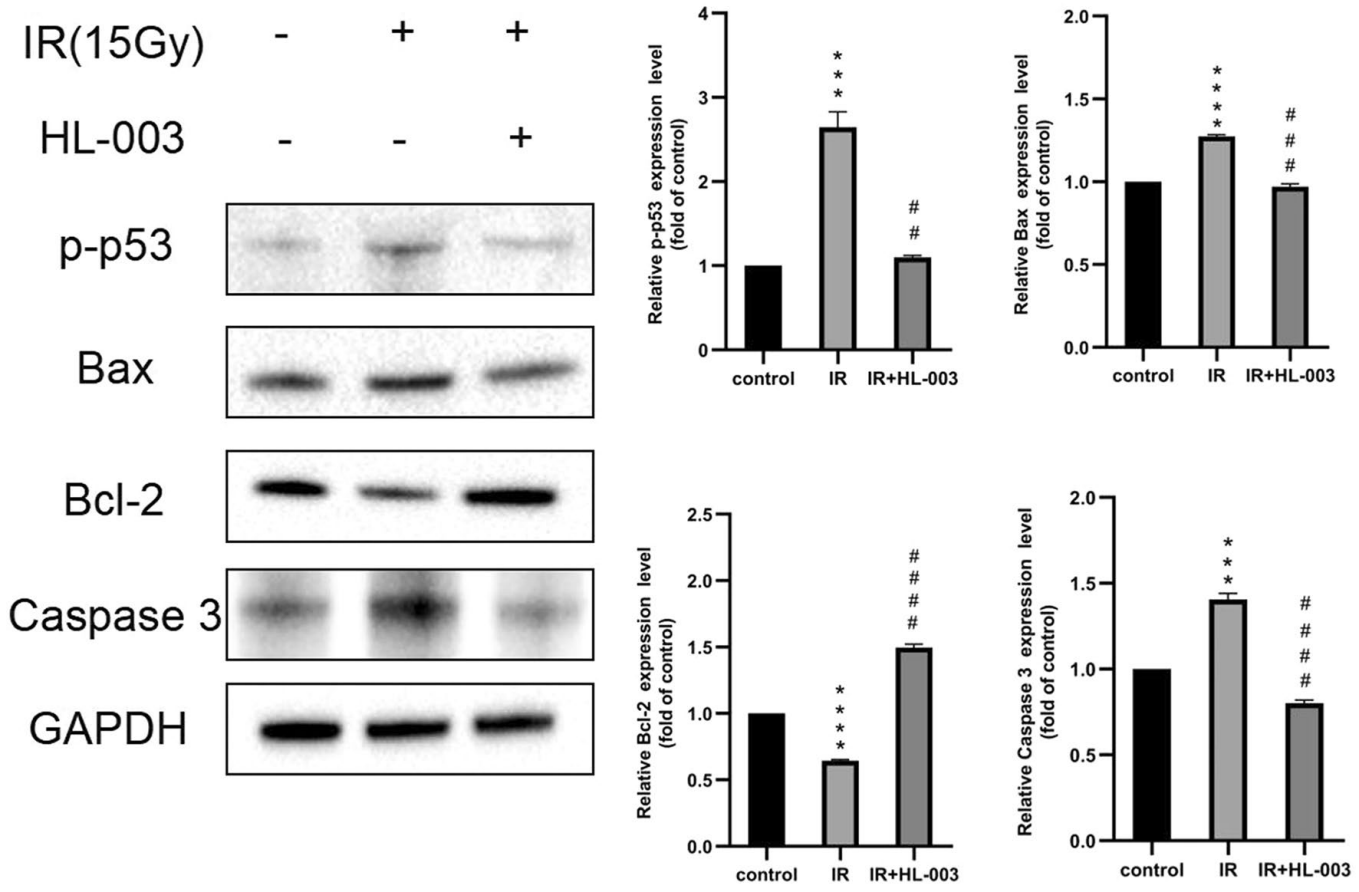
HL-003 suppresses apoptotic signaling pathway. Western blot analysis of p-p53, Bax, Bcl-2 and caspase 3. Relative quantitative analysis is shown. Each bar shows the mean ± SEM; ***p < 0.001, ****p < 0.0001, compared with the control group; ^##^p < 0.01, ^###^p < 0.001, ^####^p < 0.0001, compared with the IR group (n = 6 per group).

To further clarify how HL-003 regulates cell apoptosis, we used immunohistochemistry and western blotting to detect the expression of apoptosis-related proteins. In the IR group, the levels of caspase 9 in tissue were significantly higher than those in the control group. In the IR + HL-003 group, the levels of caspase 9 decreased significantly compared to those in the IR group (Fig. 5b,d). In addition, irradiation-induced expression levels of Bax and caspase 3 were higher than those in the control group, and irradiation-induced expression levels of Bcl-2 were significantly decreased (Fig. 6). However, treatment with HL-003 dramatically reduced the expression of Bax, caspase 3, and p-p53, but increased the expression of Bcl-2. These results indicate that HL-003 could play a positive role in alleviating IR damage by regulating the apoptotic signaling pathway.

## Discussion

Xerostomia is one of the most common complications of radiotherapy in patients with HNC, and other adverse reactions include dysphagia, osteoradionecrosis and acute mucositis^24^. Irradiation can cause structural and functional damage to acini, ducts, nerves, blood vessels, and lymphatic vessels of the salivary glands, ultimately resulting in reduced salivary secretion^25^. Xerostomia can lead to glossitis, oral candidiasis, and dental caries. The main treatments for xerostomia include the use of drugs such as pilocarpine to stimulate salivary secretion^26^, cell therapy to regenerate salivary gland tissue^27,28^, and the use of cytokines^29^ or antioxidants^13,17^. Therefore, preventive measures are key to preventing xerostomia, and it is crucial to find an effective radioprotector.

Our research group independently developed HL-003, which is a strong antioxidant with a strong free radical scavenging ability. Studies have shown that HL-003 can protect the intestine from radiation damage^21^. Hence, we hypothesized that HL-003 could effectively prevent salivary gland damage after radiotherapy. This study mainly explored the protective effect of HL-003 on salivary gland dysfunction after radiotherapy, described the internal connection between ionizing radiation damage and oxidative stress, DNA damage, and cell apoptosis through various experiments, and evaluated the radiation protective effect of HL-003.

One sign of radiation-induced salivary gland dysfunction is the reduced salivary flow rate^12^. In this study, we observed that the SFR was significantly higher in the IR + HL-003 and IR + amifostine groups than in the IR group (Fig. 1). This indicates that HL-003, comprising the same effect as amifostine, can protect the salivary glands and maintain their function. IR causes damage to the salivary gland structure, mainly manifested as a reduction in acinar number, acinar atrophy and vacuolization, duct ectasia, and capillary congestion^14^. Several pyknotic nuclei were observed in acinar cells, and inflammatory infiltration and focal fibrosis were observed in the salivary glands^15^. In our study, cytoplasmic vacuolization and few zymogen granules were observed in the IR group, while the lobule structure in the HL-003 and amifostine treatment groups was clear, and had fewer vacuoles (Fig. 2a). These results indicate that HL-003 may prevent radiation-induced structural changes in the salivary glands. Since the mechanism of amifostine has been described in relevant study^30^, we will focus on the radiation protection mechanism of HL-003 in the following experiments. As a marker of salivary epithelial cells, AQP-5 is mainly distributed in the secretory acinar cells of the parotid, submandibular, and sublingual glands, and plays an important role in the process of salivary secretion^31^. Compared with normal mice, AQP-1 and AQP-4 knockout mice showed no obvious changes in salivary volume and composition, but AQP-5 knockout mice had significant defects^32^. The expression of AQP-5 in rats decreased after IR, suggesting that AQP-5 may be involved in the pathological process of radiation-induced xerostomia^33^. Our results showed that the AQP-5 levels in the IR group were significantly lower than those in the control group, whereas the AQP-5 levels did not decrease in the HL-003 treatment group (Fig. 2b).

IR interacts with body the components through direct or indirect actions. Indirect action means that IR causes water to decompose and produce a large amount of ROS. ROS attacks unsaturated fatty acids in biofilms, resulting in lipid peroxidation, and promote a large amount of MDA production. MDA reflects the degree of oxidative stress damage in a certain extent^34^. Studies have shown that the sharp decrease in SFR in mice after irradiation is due to excessive ROS production^13^. Therefore, we explored the effect of HL-003 on oxidative stress. Our current data showed that in IR-exposed salivary gland tissues, T-AOC levels were significantly downregulated, and the MDA concentration were upregulated. In contrast, HL-003 treatment significantly improved the level of T-AOC (Fig. 3a) and reduced the MDA concentration (Fig. 3b). Previous experiments used the DPPH method to evaluate the antioxidant capacity of HL-003 in vitro^21^. Our results prove that HL-003 has strong antioxidant activity, which is similar with earlier observations. To explore how HL-003 regulates the oxidative stress, we examined the expression of ROS-related proteins. Nicotinamide adenine dinucleotide phosphate oxidases are widely distributed on the surface of cell membranes, and their activation depends on the regulation of upstream cytokines or cell matrix breakdown products^35^. NOX4 is an important subtype member of the NADPH oxidase family and is expressed in endothelial cells and vascular smooth muscle cells. NOX4 is involved in the production of ROS, membrane lipid peroxidation, inflammatory responses, and cell apoptosis^36^. In this study, NOX4 was overexpressed in the IR group, and HL-003 reversed the expression of NOX4 (Fig. 3c,d). Superoxide dismutase and 8-hydroxy-2’-deoxyguanosine are currently recognized biological markers that reflect the level of oxidative stress in vivo^37^. SOD is a kind of antioxidant enzyme that can scavenge free radicals and is used to maintain the balance of ROS in the body. However, 8-OHdG is the product of DNA attacked by ROS, which can be used to evaluate the severity of oxidative damage. Immunohistochemistry showed that in the IR + HL-003 group, the levels of SOD2 were increased, and the levels of 8-OHdG were decreased (Fig. 3e,f). These phenomena may be related to the antioxidant properties of HL-003, which indirectly reduces ROS levels and alleviates the oxidative stress response.

Ionizing radiation and oxidative stress can cause DNA damage and cell apoptosis^9,10^. It is established that γ-H2AX is a biomarker used to detect DSBs^23^. We observed that the γ-H2AX levels in radiation-induced mice were decreased after HL-003 treatment, suggesting that HL-003 can reduce DNA damage by inhibiting oxidative stress (Fig. 4). TUNEL assay results confirmed that HL-003 effectively inhibited cell apoptosis (Fig. 5a,c). In addition, excessive ROS and DNA damage can activate p53. As a pro-apoptotic molecule, p53 is not only involved in DNA damage and repair, but can also guide the transcription and translation of theBcl-2 family, and participate in cell apoptosis^38,39^. Bcl-2 is a proto-oncogene that inhibits apoptosis, and p53 can inhibit the expression of Bcl-2 and activate the pro-apoptotic factor Bax, which is an antagonist of Bcl-2. The transfer of Bax from the cytoplasm to the mitochondrial outer membrane changes the permeability of the mitochondrial outer membrane and induces the release of cytochrome c (Cyt c). Cyt c combines with another apoptotic factor, Apaf-1, to form an apoptosome that recruits initiator caspases (caspase 9) in the cytoplasm. Activated caspase 9 further acts on the effector caspases (caspase 3) to induce apoptosis^40,41^. We found that p-p53 levels in irradiated mice treated with HL-003 were downregulated (Fig. 6). This may be related to radiation-induced ROS production. Activation of p53 further affects the apoptosis signaling pathway. In the IR group, the expression of Bax, caspase 3, and caspase 9 was upregulated, whereas the Bcl-2 levels were decreased (Fig. 6). However, HL-003 treatment significantly reversed Bax/Bcl-2 signaling, as well as the expression of caspase 3 and caspase 9 in the salivary gland tissue (Fig. 6). All these changes indicated that HL-003 could reduce radiation-induced DNA damage and oxidative stress, thereby inhibiting cell apoptosis through the p53 pathway.

## Conclusions

This study demonstrates that HL-003 administration in mice protects against radiation-induced salivary gland damage. HL-003 treatment significantly decreased oxidative stress and reduced cell apoptosis in the salivary glands. Therefore, HL-003 is a potentially effective radioprotector that alleviates the side effects of salivary gland radiotherapy.

## Supplementary Information

Supplementary Information.

## Data Availability

All data included in this study are available upon request by contact with the corresponding author.
