# Supplementary material for: Radioprotective effects and mechanism of HL-003 on radiation-induced salivary gland damage in mice
Source: Sci Rep. 2022 May 19;12:8419. doi: 10.1038/s41598-022-12581-y (PMC9120142; doi:10.1038/s41598-022-12581-y)
Supplement: Supplementary file 1 — Supplementary Information. [file 41598_2022_12581_MOESM1_ESM.pdf]

Radioprotective effects and mechanism of HL-003 on radiation-induced salivary gland damage in mice

Jingming Ren,[1] Rong Huang,[2,3] Yanjie Li,[2,3] Ruiyang Chen,[2,3] Hongqi Tian,[1,4,*] and Chenlu Liu[2,3,*]

1 Tianjin Key Laboratory of Radiation Medicine and Molecular Nuclear Medicine, Institute of Radiation Medicine, Peking Union Medical College and Chinese Academy of Medical Science, Tianjin, 300192, China.

2 Department of Oral Medicine, Tianjin Stomatological Hospital, School of Medicine, Nankai University, Tianjin 300041, China.

3 Tianjin Key Laboratory of Oral and Maxillofacial Function Reconstruction, Tianjin 300041, China.

4 KeChow Pharma, Inc., 201203, Shanghai, China.

Supplementary Materials

Supplementary table 1: The changes of weight in mice (g). [a]

	Day-2	Day0	Day3	Day5	Day7	Day9	Day12
control	23.00±0.75	22.90±0.94	22.77±0.90	22.55±0.05	23.05±1.21	23.20±0.33	23.10±1.02
IR	22.60±1.29	23.14±1.15	22.94±0.98	22.57±1.53	23.65±1.34	23.68±1.38	23.76±1.44
IR + HL-003	22.60±0.89	23.00±0.97	21.63±0.87	22.66±1.07	23.24±1.21	23.74±1.11	23.67±1.19
IR + Amifostine	22.53±1.09	23.01±0.82	22.57±1.02	22.95±1.12	23.88±1.18	23.93±1.10	24.35±0.84

[a] The data are represented as the mean ± SEM (n=6).

Supplementary table 2: The changes of water intake in mice (mL/d/mice). [a]

	Day-2	Day0	Day3	Day5	Day7	Day9	Day12
control	4.16±0.93	4.58±0.24	4.58±0.61	4.38±0.67	5.00±0.17	6.16±0.78	6.75±0.25
IR	4.00±0.46	4.16±0.44	4.75±0.27	5.08±0.19	5.33±0.45	6.25±0.22	6.67±0.67
IR + HL-003	4.28±0.33	4.42±0.83	4.64±0.11	5.07±0.53	6.00±0.67	5.86±0.49	6.85±0.55
IR + Amifostine	4.33±0.17	4.83±0.10	5.00±0.31	5.32±0.38	5.33±0.33	5.92±0.63	6.67±0.82

[a]The data are represented as the mean ± SEM (n=6).

Supplementary table 3: The changes of food intake in mice (g/d/mice). [a]

	Day-2	Day0	Day3	Day5	Day7	Day9	Day12
control	3.03±0.23	3.26±0.67	4.18±0.32	3.26±0.61	4.25±0.17	4.12±0.18	3.45±0.03
IR	2.38±0.05	3.30±0.15	3.35±0.78	4.79±0.17	4.42±0.79	3.00±0.38	3.70±0.84
IR + HL-003	3.82±0.42	3.41±0.42	3.80±0.12	4.42±0.92	4.83±0.28	3.10±0.29	3.01±0.47
IR + Amifostine	3.08±0.33	3.77±0.67	4.06±0.33	3.62±0.08	4.36±0.25	2.93±0.53	3.68±0.18

[a]The data are represented as the mean ± SEM (n=6).

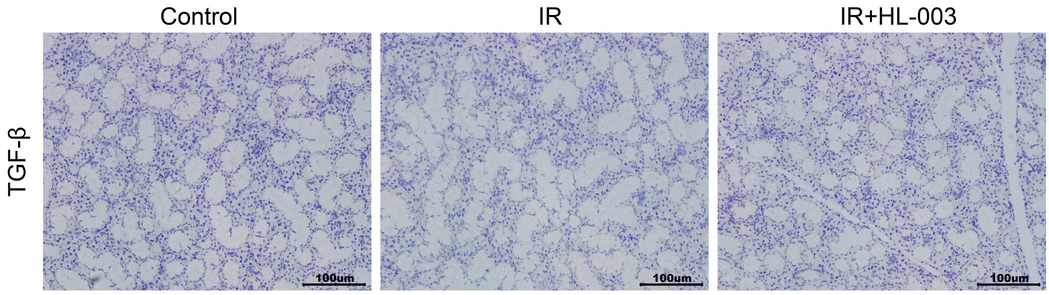

Supplementary figure 1. HL-003 may not affect TGF-β in salivary gland tissue. Scale bars represent 50μm.

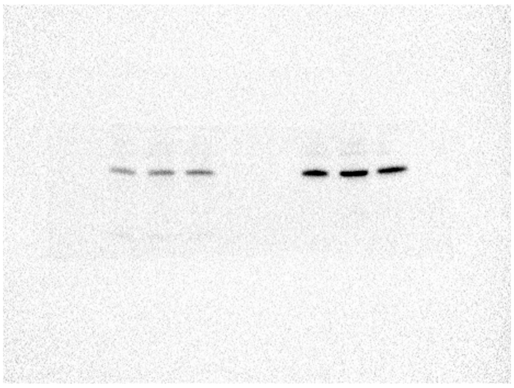

Supplementary figure 2. Western blot analysis of Bax.

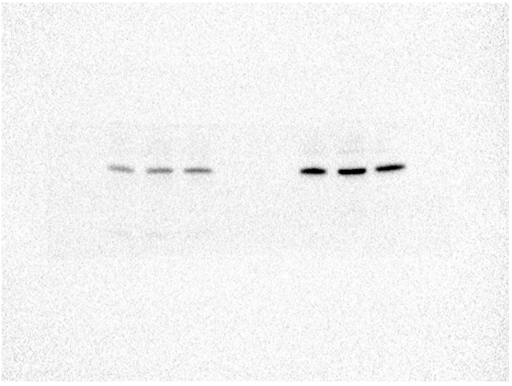

Supplementary figure 3. Western blot analysis of Bax.

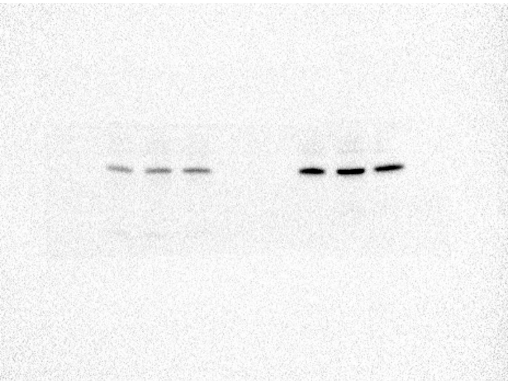

Supplementary figure 4. Western blot analysis of Bax.

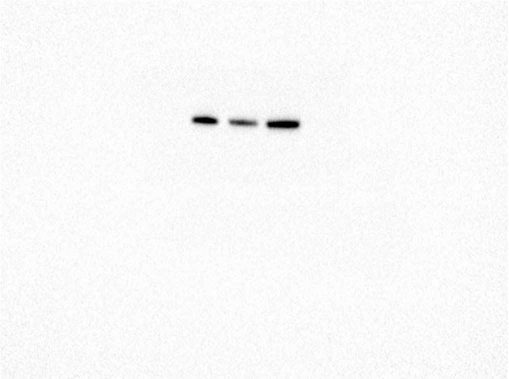

Supplementary figure 5. Western blot analysis of Bcl-2.

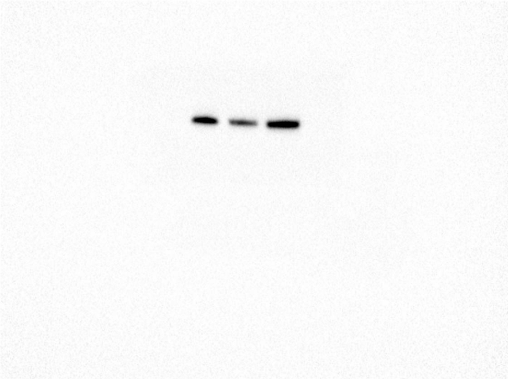

Supplementary figure 6. Western blot analysis of Bcl-2.

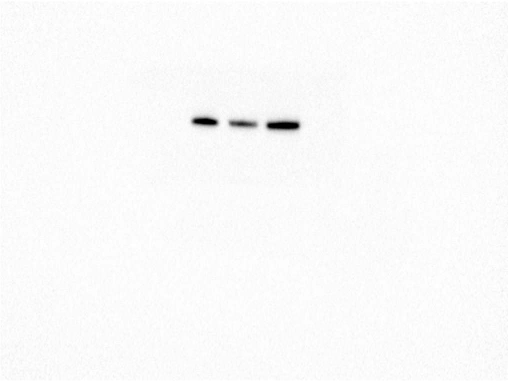

Supplementary figure 7. Western blot analysis of Bcl-2.

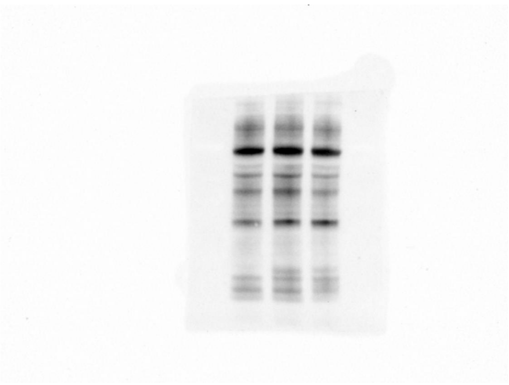

Supplementary figure 8. Western blot analysis of caspase-3.

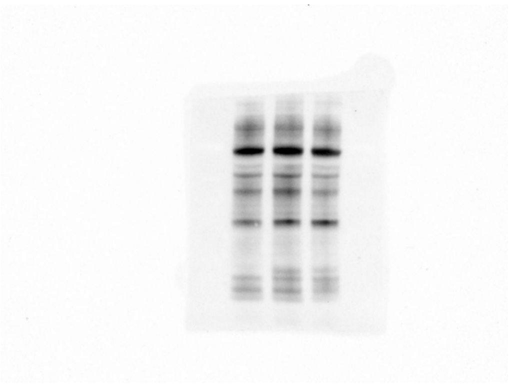

Supplementary figure 9. Western blot analysis of caspase-3.

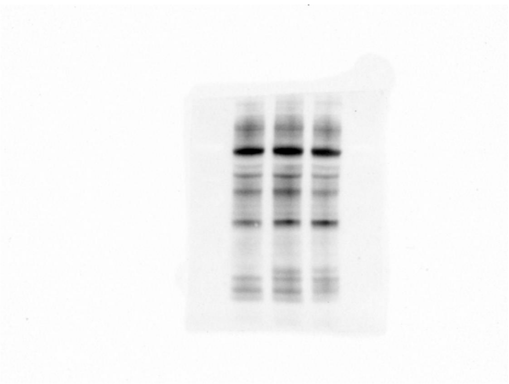

Supplementary figure 10. Western blot analysis of caspase-3.

Supplementary figure 11. Western blot analysis of GAPDH.

Supplementary figure 12. Western blot analysis of GAPDH.

Supplementary figure 13. Western blot analysis of GAPDH.

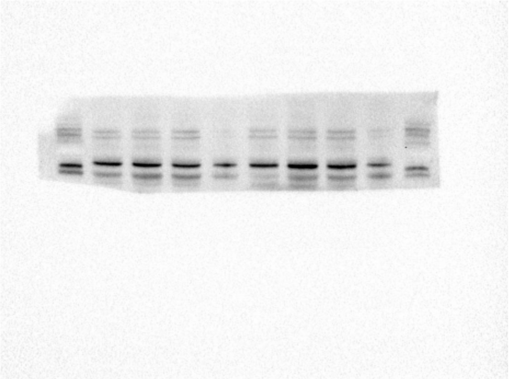

Supplementary figure 14. Western blot analysis of NOX4.

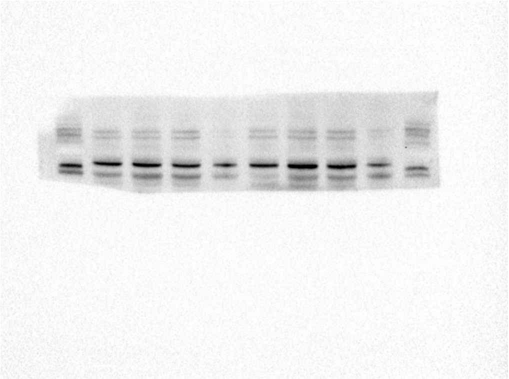

Supplementary figure 15. Western blot analysis of NOX4.

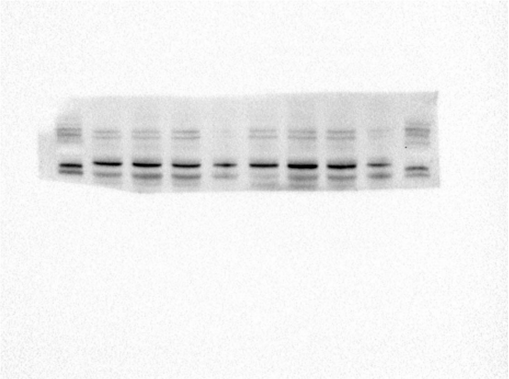

Supplementary figure 16. Western blot analysis of NOX4.

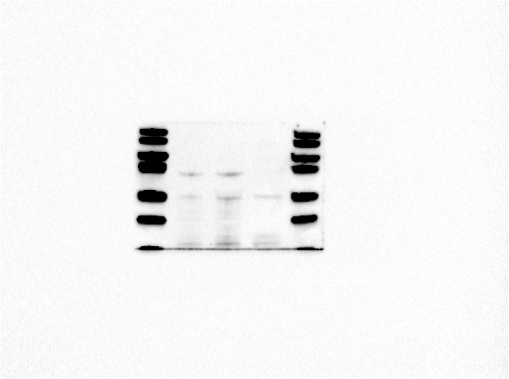

Supplementary figure 17. Western blot analysis of p-p53.

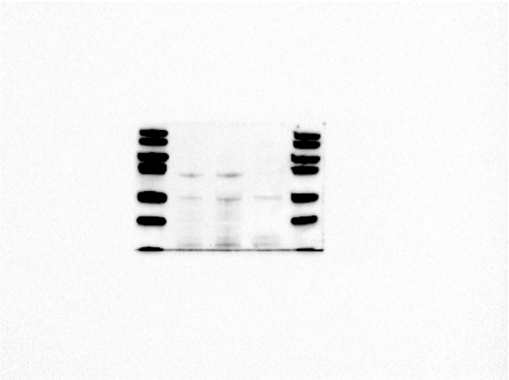

Supplementary figure 18. Western blot analysis of p-p53.

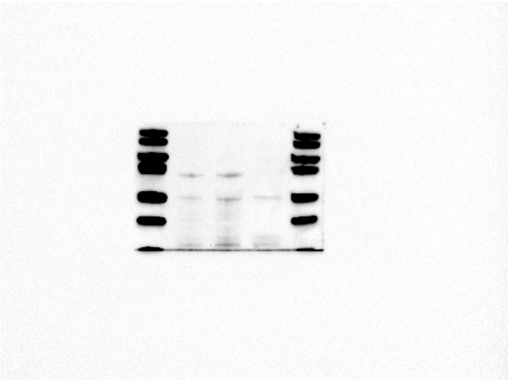

Supplementary figure 19. Western blot analysis of p-p53.
